# Density Functional Theory description of the order-disorder transformation in Fe-Ni

**DOI:** 10.1038/s41598-019-44506-7

**Published:** 2019-06-03

**Authors:** Li-Yun Tian, Henrik Levämäki, Olle Eriksson, Kalevi Kokko, Ágnes Nagy, Erna Krisztina Délczeg-Czirják, Levente Vitos

**Affiliations:** 1 Applied Materials Physics, Department of Materials Science and Engineering, Royal Institute of Technology, Stockholm, SE-100 44 Sweden; 2 Department of Physics and Astronomy, Division of Materials Theory, Uppsala University, Box 516, SE-751 20 Uppsala, Sweden; 3 School of Science and Engineering, Örebro University, Örebro, Sweden; 4 Department of Physics and Astronomy, University of Turku, FI-20014 Turku, Finland; 5 Turku University Centre for Materials and Surfaces (MatSurf), Turku, Finland; 6 Department of Theoretical Physics, University of Debrecen, H-4010 Debrecen, Hungary; 7 Research Institute for Solid State Physics and Optics, Wigner Research Center for Physics, Budapest, H-1525 Hungary

**Keywords:** Metals and alloys, Electronic structure

## Abstract

The thermodynamic ordering transformation of tetragonal FeNi system is investigated by the Exact Muffin-Tin Orbitals (EMTO) method. The tetragonal distortion of the unit cell is taken into account and the free energy is calculated as a function of long-range order and includes the configurational, vibrational, electronic and magnetic contributions. We find that both configurational and vibrational effects are important and that the vibrational effect lowers the predicted transformation temperature by about 480 K compared to the value obtained merely from the configurational free energy. The predicted temperature is in excellent agreement with the experimental value when all contributions are taken into account. We also perform spin dynamics calculations for the magnetic transition temperature and find it to be in agreement with the experiments. The present research opens new opportunities for quantum-mechanical engineering of the chemical and magnetic ordering in tetrataenite.

## Introduction

Permanent magnet materials have attracted particular attention due to their applications in modern life, *e.g*. for transformation of mechanical energy to electricity. These magnets exhibit large uniaxial magnetic anisotropy and high saturation magnetization. There is however the problem with the cost as they mostly include expensive rare-earth elements^1,2^. Hence there is a great need, in many technologies, of advanced rare-earth-free permanent magnets that can maintain the great performance of rare-earth magnets, or at least to have equal price - performance capability^3^.

It has been confirmed that tetragonal L1_0_ FeNi (tetrataenite) is a promising permanent magnet with large uniaxial magnetic anisotropy, K_*u*_ = 7.0 × 10^6^ erg cm^3^ ^4^, and high Curie temperature (T_c_ ≥ 823 K)^5^. These excellent magnetic characteristics are absent in the disordered phase. Unfortunately, the L1_0_ crystal structure of FeNi is extremely challenging to fabricate as it has a low chemical order-disorder transition temperature of T_od_ ≈ 593 K^6^, which is too low to enable efficient growth of this phase. Forming the tetrataenite phase requires cooling times of the order of millions of years due to the slow diffusion of Fe and Ni atoms below T_od_. As a consequence, the tetrataenite compound naturally occurs only in meteorites, whose age exceeds these very slow formation times. Recently, interest in L1_0_ FeNi has intensified after researchers invented a new and practicable technique to form tetragonal FeNi with a high degree of order. This new technique works through nitrogen insertion and topotactic extraction^7,8^.

The simulation of phase transformations of alloys is one of the fundamental problems in the modern condensed matter theory. Despite years of efforts and exploration of practical theoretical models describing these transformations, the properties of alloys still remain one of the least explored issues in first-principles theory. Concerning tetrataenite, some *ab initio* descriptions of the transformation temperature have been reported in the literature. Mohri *et al*. performed a first-principles study of the L1_0_-disorder phase equilibria of the FeNi system and confirmed that the transition temperature is about 520 K without thermal vibrational effect^9,10^. The calculated transition temperature was found to further decrease by about 40 K when the vibration effects were taken into account. These previous calculations were carried out by Full potential Linear Augmented Plane Wave method (FLAPW)^11^.

In a recent work, Ekholm *et al*.^12^ provided an alternative solution to obtain the transition temperature for permalloy. The partial disordered local moment (PDLM) approach was adopted to describe the effect of magnetic degrees of freedom, and the configurational entropy was included using an Ising-type Hamiltonian. That model is a simple way to get the “correct” prediction while the other entropy contributions from the vibrational (phonon − *S*_vib_), electronic (*S*_el_) and magnetic (*S*_mag_) effects are ignored. More recently Tetsuo *et al*.^10^ found that the thermal vibration effects make a significant contribution to the transition temperature of L1_0_-disorder phase of FeNi using a Cluster Variation Method combined with FLAPW total energy calculations. Furthermore, a magnetic Cluster Expansion model was developed to describe a broad range of magnetic and structural transformation effects in FeNi alloys^13^. In the present work, we aim to provide a unified theoretical approach based on Density Functional Theory (DFT) to understand and predict the formation of the tetrataenite phase. This will prompt us to develop an effective and accurate method for modeling fully or partially ordered and disordered alloys.

## Results

**Figure 1:**
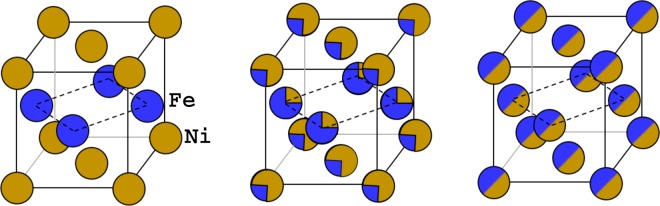
Schematic of FeNi alloy for L1_0_ ordered phase (*η* = 1.0, left), partially ordered phase (*η* = 0.5, middle), and disordered phase (*η* = 0.0, right).

For tetragonal FeNi with L1_0_ structure, Ni atoms are located at the (0, 0, 0) and ($\frac{1}{2}$, $\frac{1}{2}$, 0) sublattices and Fe atoms occupy the (0, $\frac{1}{2}$, $\frac{1}{2}$) and ($\frac{1}{2}$, 0, $\frac{1}{2}$) sublattices, as illustrated in Fig. 1 (left). For the partial ordering of FeNi, if a sublattice is occupied by different atomic species, we assume that these atoms distribute randomly on the sublattice as shown in Fig. 1 (middle and right). The random distribution of atoms is taken into account using the coherent potential approximation (CPA)^14,15^. The degree of disorder in the sublattice can be described by the long-range order (LRO) parameter *η*. The unit cell of L1_0_ FeNi includes four sublattices represented as Ni_2_Fe_2_. When considering the degree of LRO as changing the compositions in Ni and Fe layers, the formula unit can be represented with (Ni_1−*x*_Fe_*x*_)_2_(Fe_1−*x*_Ni_*x*_)_2_. Hence, the degree of LRO may be expressed as *η* = 1 − 2*x* (0 ≤ *x* ≤ 0.5). The natural tetrataenite compound of the meteorites has a small tetragonal distortion. Here the c/a ratio of L1_0_ FeNi is obtained by relaxing the unit cell. Our calculated *c*/*a* is 1.0073 (±0.001) which is very close to the measured value 1.007^4,7^. In Table 1, we list the c/a ratios for different site occupation configurations. All calculations are performed using the *ab initio* calculations within the exact muffin-tin orbitals (EMTO) method^16–18^ briefly introduced in the Methods section.

**Table 1:** The calculated *c*/*a* ratio, bulk modulus (in GPa), total and local magnetic moments of Fe and Ni (in *μ*_*B*_/atom) in ferromagnetic FeNi, as a function of *η*.

*η*	Site occupancy	*c*/*a*	B (GPa)	*μ*_*tot*_	*μ*_*Ni*_	*μ*_*Fe*_
0.0	(*Ni*_0.5_Fe_0.5_)_2_(Fe_0.5_Ni_0.5_)_2_	1.0000	180.59	1.600	0.607	2.593
0.2	(Ni_0.6_Fe_0.4_)_2_(Fe_0.6_Ni_0.4_)_2_	1.0017	181.96	1.602	0.609	2.594
0.4	(Ni_0.7_Fe_0.3_)_2_(Fe_0.7_Ni_0.3_)_2_	1.0030	182.25	1.604	0.613	2.596
0.6	(Ni_0.8_Fe_0.2_)_2_(Fe_0.8_Ni_0.2_)_2_	1.0059	182.81	1.608	0.617	2.599
0.8	(Ni_0.9_Fe_0.1_)_2_(Fe_0.9_Ni_0.1_)_2_	1.0072	183.82	1.612	0.618	2.606
1.0	Ni_2_Fe_2_	1.0073	185.87	1.616	0.613	2.619

**Figure 2:**
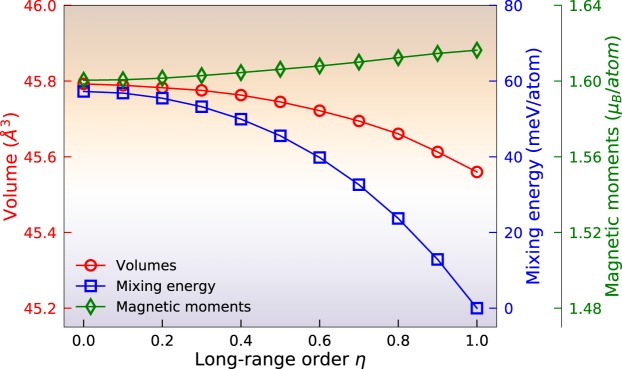
The equilibrium volumes, mixing energies (the total energy of L1_0_ FeNi is used as a reference) and magnetic moments of FeNi as a function of the order parameter *η*.

The order-disorder transformation of FeNi was investigated using LRO structure model in the ferromagnetic state. In Fig. 2, the equilibrium volumes and total energy differences Δ*E* relative to the ordered alloy are shown as a function of *η*. The equilibrium volumes of fully and partially ordered FeNi alloys are fitted by the Birch-Murnaghan equation of state^19,20^. The volumes of FeNi alloys differ by about 0.5% between the fully ordered and disordered states. This implies that the volume effect, which in this case is induced by changing the site occupations, is quite small. That is because the atomic sizes of Ni and Fe are very similar. The energy differences Δ*E* were calculated for different site occupations relative to the ordered L1_0_ FeNi configuration. As shown in Fig. 2, the value of Δ*E* decreases with increasing the ordering parameter, *η*, which means that the fully ordered FeNi configuration is more stable than the partially ordered and fully disordered phases at 0 K. In Table 1, the bulk moduli in ferromagnetic FeNi alloys are listed with different *η*. As shown in the Table, the bulk moduli change is less than 2.84% due to the small variation in the volumes.

Presented also in Table 1 and Fig. 2 are the total magnetic moments of FeNi for various choices of *η*. The total magnetic moment is increased slightly from 1.600 *μ*_*B*_ to 1.616 *μ*_*B*_ with increasing *η*. We notice that the Ni local magnetic moments remain nearly constant as a function of *η*, while the Fe local magnetic moments slightly increase. These changes do not seem to come from the volume effects, because the volume decreases as a function of *η* and usually the moments increase with increasing volume. The total magnetic moments for alternating atomic layers along the *z*-direction, increase (decrease) linearly from 1.600 (1.600) *μ*_*B*_ to 2.619 (0.613) *μ*_*B*_ with *η* as the Fe (Ni) atoms moving to their own layers in the fully ordered structure.

**Figure 3:**
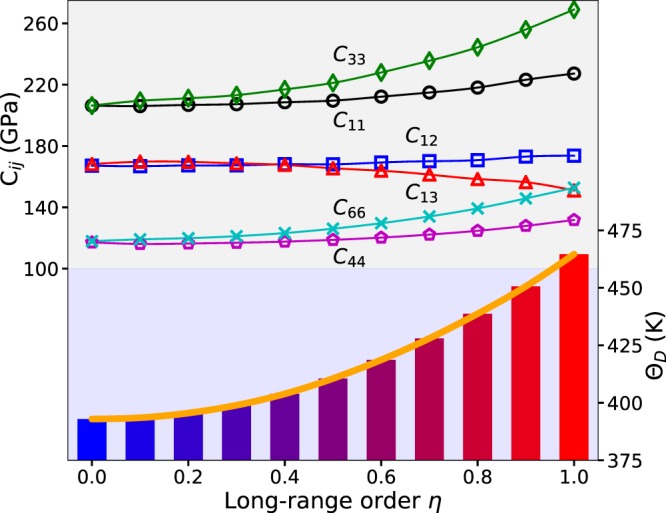
The elastic constants *C*_*ij*_ and Debye temperature Θ_*D*_ of FeNi as a function of the order parameter *η*.

In Fig. 3, six independent elastic parameters *C*_11_, *C*_12_, *C*_13_, *C*_33_, *C*_44_, and *C*_66_ are presented as a function of *η*. The elastic constants *C*_*ij*_ for a tetragonal crystal were calculated using the orthorhombic and monoclinic deformations. More details about the deformations for tetragonal crystals can be found in the ref.^21^. The single-crystal elastic constants *C*_11_, *C*_12_, *C*_13_, *C*_33_, *C*_44_ and *C*_66_ are affected by the ordering. As shown in Fig. 3, except for *C*_13_, the ordered FeNi alloy possesses higher *C*_11_, *C*_12_, *C*_33_, *C*_44_, *C*_66_ than the random and partially ordered alloys. For ordered FeNi, *C*_13_ is lower than those for the partially ordered alloys. Similar conclusions have been made for cubic Ni_3_Fe alloys^22^. The elastic Debye temperature Θ_*D*_ is determined from the polycrystalline elastic moduli (*B* and *G*) and average density *ρ*. It is found that Θ_*D*_ increases with *η* making the system elastically stiffer in the ordered case. In other words, lattice vibrations are expected to favor the disordered state against the ordered one, at elevated temperatures.

**Figure 4:**
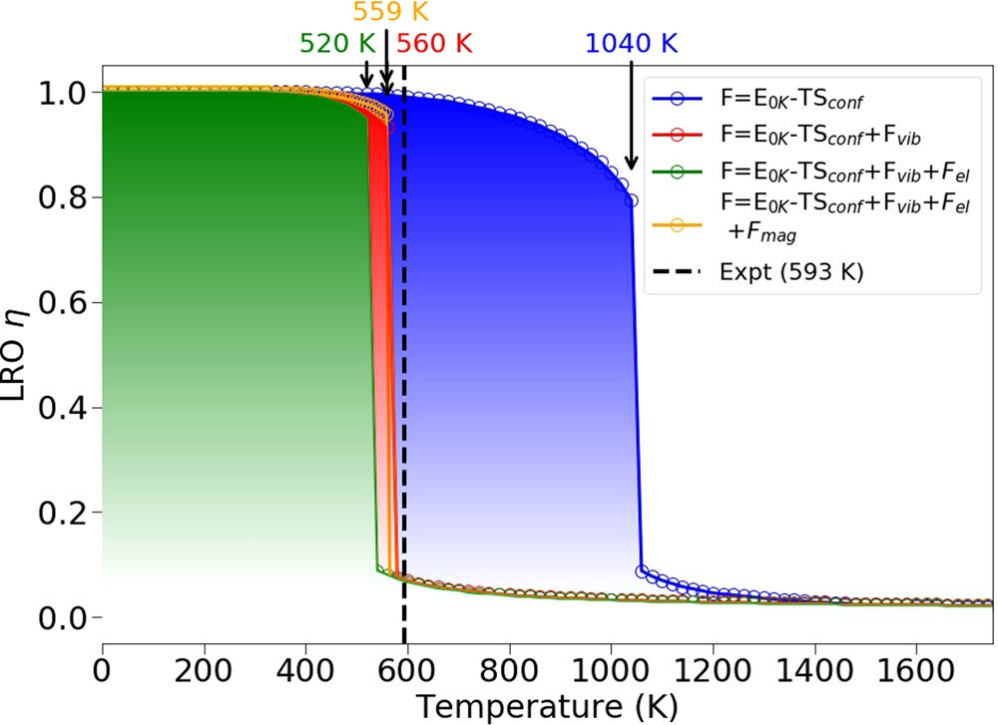
Calculated order-disorder transformation in FeNi.

The transformation between ordered and disordered phases depends on the temperature and atomic ordering *η* and is presented in Fig. 4. There is a clear first order transformation from the low-temperature ordered phase to the high-temperature disordered phase. To reflect the most interesting structural features of the transformation, we consider separately the configurational, vibrational, electronic and magnetic free energy contributions. The temperature dependent curves correspond to the free energy with and without vibrational or electronic or magnetic contributions. The predicted critical temperature T_od_ is about 1040 K with configurational contribution only, which is far from the experimental value of 593 K^6^. By including the vibrational effect, the curve shifts to lower temperature and T_od_ is reduced to about 560 K. The vibrational contribution has therefore a very large effect on T_od_ and we can conclude that the phonon contribution is an important factor affecting ordering in FeNi. The reason behind this effect will be addressed later. The electronic entropy decreases the critical temperature T_od_ to 520 K, while the magnetic entropy shows a positive effect. Actually, the electronic and magnetic contributions nearly cancel each other bringing the final T_od_ back to 559 K.

Because the total magnetic moment hardly changes as a function of *η*, the first order transition that we observe is of chemical nature. The magnetic transition temperature occurs at a higher temperature than the chemical transition. Dang *et al*. studied the chemical and magnetic order-disorder transitions of FeNi alloys using a Monte Carlo Ising model^23^. They simulated transitions in the magnetic moment and order parameter separately for magnetic and chemical effects, and also transitions that were caused by both effects acting simultaneously. Their transition temperature coming from the chemical effects only is about 620 K, which is close to our present transition temperature of 560 K, even though their simulations did not take vibrational effects into account, while the present consideration did. For the combined chemical and magnetic simulation Dang *et al*. report a LRO parameter transition temperature of about 720 K. The magnetic transition happens later at about 900 K and at the chemical transition temperature the magnetization is still close to the 0 K value, which agrees with our data, since our magnetic moments also hardly change as a function of *η*.

We have also calculated the Curie temperature *T*_*c*_ via Heisenberg exchange parameters obtained from the EMTO *ab-initio* theory. These parameters are used for Monte Carlo (MC) simulations with the UppASD program package^24^. These simulations aimed obtaining a complete picture for the material, and to evaluate if sufficiently high ordering temperatures could be obtained to render the material suitable for permanent magnet applications. Previous work shows that binary magnetic compounds are suitable to be used in the permanent magnet applications with *T*_*c*_s in the order of 600 K or higher^25^. In the present work, the *T*_*c*_ for fully order FeNi is 780 K, while the *T*_*c*_ is 630 K for fully random phase. It is noted that the high degree of chemical ordering increases the *T*_*c*_.

**Figure 5:**
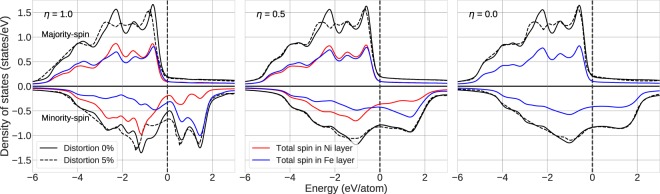
The total electronic density of states of FeNi with different long-range parameter *η*. The energy scale is defined so that the Fermi energy is zero. Dashed lines show the DOS for 5% monoclinic distortion used to derive the (*C*_11_ − 2*C*_13_ + *C*_33_) elastic parameter.

Figure 5 shows the total electronic density of states (DOS) of the valence states of FeNi. Results are shown for the ordered (*η* = 1.0), partially ordered (*η* = 0.5), and random (*η* = 0.0) cases. We find that the total majority-spin DOSes remain fairly unchanged for any value of *η*, which is because the total magnetic moments are not significantly changed during the ordering process (Table 1). It is interesting to note that while the minority spin channel becomes smooth upon disordering (a typical disordering effect) the majority spin channel DOS hardly changes. Benea *et al*. noticed the same phenomenon for their FeNi DOS that was calculated using dynamical mean field theory (DMFT)^26^. They ascribed the effect to be stemming from the fact that the minority spin 3*d* orbitals react to disordering more strongly, because the minority spin orbitals are spatially more extended.

By comparing the partial DOS on Ni and Fe layers in Fig. 5, we can reveal further details behind the calculated ordering effect on the magnetic properties. Considering the relative differences between the ordered and disordered DOS, we conclude that there exists a strong ordering effect mostly for the minority channel, which modifies the partial Ni and Fe DOS due to the changes in local chemical environment. We see that the minority spin projection of the atomic layer with atoms at *z* = 0, have a DOS that becomes less occupied when the disorder increases. For the layer with atoms at *z* = 0.5, the disorder induced smearing of the minority spin DOS means that the minority spin DOS for the Fe layer becomes more occupied. This is in line with that both Fe and Ni have more than half filled *d* orbitals or *sd* hybrid orbitals. So during disordering electrons are removed (added) to Ni (Fe) layer, respectively, mostly from (to) minority states.

The minority spin DOS at Fermi level in random state (−0.83 state/eV at *η* = 0.0) is larger than the one in the ordered one (−0.5 state/eV at *η* = 1.0), which may tentatively support the stability of the ordered tetragonal state. More importantly, there is a pseudo gap near the Fermi level in the minority DOS for the ordered state. This signals increased dynamical stability as compared to the chemically disordered alloy. Indeed, comparing the L1_0_ DOS after 5% monoclinic distortion used for the (*C*_11_ − 2*C*_13_ + *C*_33_) elastic parameter with the undistorted one, we see that the pseudogap is largely removed by symmetry lowering deformation. This gives a strong increase of the kinetic energy. No such effect is present in the disordered alloy which explains the pronounced increase of the above combined elastic parameter and in particular of the *C*_33_ upon ordering (Fig. 3).

## Conclusions

Up to now, successful *ab initio* investigations are absent for the tetragonal FeNi. In this work we focus on the phase transition of tetragonal FeNi based on the first-principles theory. We find a strong dependence of the transition temperature T_od_ on the configurational and vibrational degrees of freedom. The elastic Debye model is used to describe the vibrational contribution. The Debye temperature increases by about 71.7 K when the degree of long-range order changes from the fully random state to the ordered state. The increase of the Debye temperature is connected to the fact that the ordered state has a noticeable pseudogap in the minority-spin DOS and the random state does not. As a consequence, the order-disorder transition temperature T_od_ is decreased by about 480 K due to the vibrational effects. Our calculations yield a final T_od_ = 559 K compared to the experimental value of 593 K. The magnetic transition temperature for both ordered and disordered states are calculated to be above the chemical ordering temperature. The present research demonstrates that a careful DFT study reproduces quantitatively the order-disorder transition in Fe-Ni system and gives a theoretical platform to study alloying effects with the ultimate goal to stabilize the FeNi compound at elevated temperatures. It provides a strict theoretical basis to understand and predict the formation of the tetrataenite phase. This opens a possibility to develop an effective and accurate method for modeling tetragonal ordered forms of FeNi alloys, by considerations of ternary FeNiX based alloys.

## Methods

The first-principles calculations were performed within the exact-muffin-tin orbitals (EMTO) method^16–18^ based on Density Functional Theory^27^. The *s*, *p*, *d*, and *f* orbitals were included in the EMTO basis sets. The single-electron Kohn-Shan equations were solved by the Green’s function technique and the compositional disorder was treated using the coherent-potential approximation (CPA)^14,15^. The total energies were computed via the full charge density technique^28^. The exchange-correlation functional was approximated by the Perdew, Burke, and Ernzerhof (PBE)^29^ generalized gradient approximation. The Brillouin zone was sampled by a uniform *k*-point mesh. We found that a 21 × 21 × 21 *k* mesh gave well converged total energies for the present systems. Because the atomic radii of Fe and Ni are close to each other, the effect of the local lattice relaxation was considered to be negligible. The magnetic transition temperatures were estimated using the UppASD spin dynamics code^24^. The transition temperature was estimated from the peak of the temperature dependent susceptibility curve, which was enough for our purposes. A more accurate estimate for the transition temperature could be obtained by using the Binder cumulant method. The simulation cell size was 20 × 20 × 20 (32 000 atoms). The initialization phase was run for 10 000 iterations and after that the measurement phase was run for 20 000 iterations. The number of ensembles, over which the measurements are averaged to cancel some of the random measurement errors, was chosen to be five.

By considering the effects of LRO as a function of *η*, the free energies of ordered, partially ordered and disordered FeNi phases were expressed as

1 $$F(V,T,\eta )\,=\,{E}_{0K}(V,\eta )\,-\,T{S}_{conf}(\eta )\,+\,{F}_{vib}(V,T,\eta )\,+\,{F}_{el}(V,T,\eta )\,+\,{F}_{mag}(V,\,T,\,\eta )$$

where *E*_0*K*_ is the internal energy per unit cell at 0 K, *S*_*con f*_ is the configurational entropy, *F*_*vib*_, *F*_*el*_ and *F*_*mag*_ are the vibrational, electronic and magnetic free energies, respectively. According to the static Concentration Waves method^30^, the configurational entropy of L1_0_ structures was described as a function of LRO parameter *η* in the form

2 $$\begin{array}{ccc}{S}_{conf}(\eta ) & = & \frac{1}{N}[2\times (c+\frac{1}{2}\eta )\times \,{\rm{l}}{\rm{n}}(c+\frac{1}{2}\eta )\\  &  & +\,2\times (c-\frac{1}{2}\eta )\times \,{\rm{l}}{\rm{n}}(c-\frac{1}{2}\eta )\\  &  & +\,2\times (1-c-\frac{1}{2}\eta )\times \,{\rm{l}}{\rm{n}}(1-c-\frac{1}{2})\\  &  & +\,2\times (1-c+\frac{1}{2}\eta )\times \,{\rm{l}}{\rm{n}}(1-c+\frac{1}{2}\eta )].\end{array}$$

Here *c* corresponds to the atomic fraction of the solute. Detailed information about the approach can be found in ref.^30^.

The vibrational contribution to Helmholtz free energy, *F*_*vib*_(*V*, *T*, *η*) = *E*_*vib*_ (V, T, η)− *TS*_*vib*_(V,T,η), was described by Debye model with the Debye temperatures determined by the tetragonal elastic parameters. It has been proved that the Debye model depicts well the thermodynamic properties of metals and their alloys^22,31^. The electronic contribution to free energy was estimated by ${F}_{el}(V,T,\eta )\approx -\frac{1}{2}T{S}_{el}(V,T,\eta )\approx -\frac{2{\pi }^{2}}{3}{k}_{B}^{2}{T}^{2}{N}_{el}({\varepsilon }_{F},V,\eta )$, where electronic density of state *N*_*el*_(*ε*_*F*_, V, *η*) is approximated to be constant in the neighborhood of the Fermi level *ε*_*F*_. The magnetic contribution to free energy, ${F}_{mag}(V,T,\eta )=-T{S}_{mag}(V,T,\eta )=-T\frac{{\rm{\partial }}\langle {H}_{mag}(V,\eta )\rangle }{{\rm{\partial }}T}$, and Heisenberg exchange Hamiltonian ${H}_{mag}(V,\eta )=-\frac{1}{2}{\sum }_{i\ne j}{J}_{ij}(V,\eta ){\mu }_{i}(V,\eta ){\mu }_{j}(V,\eta ){\hat{e}}_{i}{\hat{e}}_{j}$ where *J*_*ij*_ (V, η) is the Heisenberg exchange interaction between atoms *i* and *j*, and *μ*_*i*_(V, η) and *μ*_*j*_(V, η) are the local magnetic moments on sites *i* and *j*.

The LRO parameter was obtained as a function of temperature by numerically minimizing the free-energy as a function of *η*, *viz*

3 $$\frac{\partial F}{\partial \eta }=0$$

for a range of different temperatures. The order-disorder temperature *T*_od_ was then obtained by computing ∂*η*/∂*T* and checking at what temperature the discontinuity appears (Fig. 4).
